# Female rule in lemurs is ancestral and hormonally mediated

**DOI:** 10.1038/srep09631

**Published:** 2015-05-07

**Authors:** Joseph M. A. Petty, Christine M. Drea

**Affiliations:** 1 University Program in Ecology, Duke University, Durham, NC 27708 USA; 2 Department of Evolutionary Anthropology, Duke University, Durham, NC 27708 USA; 3 Department of Biology, Duke University, Durham, NC 27708 USA

## Abstract

Female social dominance (FSD) over males is unusual in mammals, yet characterizes most Malagasy lemurs, which represent almost 30% of all primates. Despite its prevalence in this suborder, both the evolutionary trajectory and proximate mechanism of FSD remain unclear. Potentially associated with FSD is a suite of behavioural, physiological and morphological traits in females that implicates (as a putative mechanism) ‘masculinization’ via androgen exposure; however, relative to conspecific males, female lemurs curiously show little evidence of raised androgen concentrations. By observing mixed‐sex pairs of related *Eulemur* species, we identified two key study groups ‐‐ one comprised of species expressing FSD and increased female scent marking, the other comprised of species (from a recently evolved clade) showing equal status between the sexes and the more traditional pattern of sexually dimorphic behaviour. Comparing females from these two groups, we show that FSD is associated with more masculine androgen profiles. Based on the widespread prevalence of male‐like features in female lemurs and a current phylogeny, we suggest that relaxation of hormonally mediated FSD emerged only recently and that female masculinization may be the ancestral lemur condition, an idea that could revolutionize our understanding of the ancient socioecology and evolution of primate social systems.

In most mammals, males are the more aggressive sex^1^ and, in hierarchical societies, often dominate females^2^, but in strepsirrhine (or lemuriform) primates, female social dominance (FSD) over males is the norm^3^,^4^. Although strepsirrhines account for a large proportion of extant primate diversity^5^, they remain relatively understudied; accordingly, we still understand little about the evolution or mechanism of their unusual social organization. Importantly, aggressively mediated FSD across taxa can be associated with other exceptional traits in females, relative to conspecific males (including vigorous play, pronounced scent marking, enhanced body size, delayed puberty, and male‐like external genitalia), that reduce, eliminate or even reverse typical sex differences^6^,^7^. According to the theory of mammalian sexual differentiation^8^,^9^,^10^,^11^, this suite of traits in females implicates hormonal masculinization, presumably via appropriately timed androgen exposure. If so, endocrine markers should be evident both prenatally (as mediators of permanent, organizational effects) and in adulthood (as mediators of transient, activational effects). Here, we suggest that the prevalence and co‐occurrence of these traits across lemurid taxa^12^,^13^, coupled with our novel evidence of neuroendocrine covariation within the *Eulemur* clade, point to FSD as a likely ancestral, hormonally mediated condition (figure 1)^1^,^14^,^15^,^16^,^17^,^18^,^19^,^20^,^21^,^22^,^23^,^24^.

In the best known cases of FSD – displayed by the spotted hyena (*Crocuta crocuta*)^6^ and the ring‐tailed lemur (*Lemur catta*)^7^ – testosterone (T: 17β‐hydroxyandrost‐4‐en‐3‐one) and its precursor, androstenedione (A_4_: androst‐4‐ene‐3,17‐dione), are either dramatically or moderately elevated during pregnancy, providing a basis for organizational effects on developing offspring; otherwise, T in adulthood, rather puzzlingly, follows the typical mammalian pattern of much reduced concentrations in females relative to males^23^,^29^,^30^,^31^,^32^,^33^, raising an enduring question about activational effects. A better understanding of the relevance of adult androgen concentrations in activating FSD could derive from a comparative study of closely related species in which the females express varying degrees of masculinization; however, such an approach has been hindered by the fact that both key species – belonging to *Crocuta* and *Lemur*, respectively – are the sole extant members of their genera.

To identify a putative, masculine endocrine signature of FSD in adulthood and improve our understanding of the mediators of female aggression, we extended our studies to the sister clade of *Lemur*, the *Eulemur* (figure 1). As with other Malagasy primates, most *Eulemur* species show aggressively mediated FSD^24^,^25^,^26^,^27^. Remarkably, however, this genus also contains a relatively recently evolved clade, comprising the *E. fulvus* species, in which females purportedly share equal status with males^26^,^28^. In this first‐ever comparative behavioural and adult neuroendocrine study of FSD, we examined six *Eulemur* species, four characterized by FSD and two characterized, putatively, by egalitarianism, which we collapsed into two study groups.

Interestingly, all *Eulemur* females are characterized morphologically by masculinized genitalia (in that the clitoris is elongated, pendulous, and partially traversed by the urethra) and by prominent anogenital glands that often are more elaborate than are those of conspecific males (figure 2)^12^,^34^. Despite similarities in female glandular morphology across *Eulemur* species, females from species with FSD (hereafter ‘dom‐*E*’ females) produce olfactory signals in adulthood that are more chemically complex than are those of conspecific males, whereas females from species that are putatively egalitarian and show sexual ‘co‐dominance’ (hereafter ‘co‐*E*’ females) do not^34^. Increased scent‐signal complexity in dom‐*E* females relative to co‐*E* females might be indicative of an unusually important role in the former for scent marking, which would be consistent with female behavioural masculinization. Ideally, a potential mechanism of FSD would explain the entire suite of masculinized characteristics in females, as well as the observed patterns of individual, sexual, and species variation^13^. Notably, the similarity in morphological traits across *Eulemur* species might implicate uniform organizational effects in females via exposure to prenatal steroids, whereas the disassociation between masculinized morphological and behavioural traits across *Eulemur* species could be explained by variability in adult, activational effects of steroids.

## Results and Discussion

Because the attribution of either FSD or sexual co‐dominance in *Eulemur* derives from studies in which researchers had measured various outcomes (e.g. feeding priority versus agonistic ‘wins’), under varying socioecological conditions, both in captivity and in the wild^3^,^4^,^26^,^28^,^35^, our first aim was to validate these categorizations using the same criteria for all species, evaluated under constant social (i.e., pair‐living) and environmental conditions. Indeed, our comparative behavioural data were consistent with prior categorizations, notably with the presence of ‘masculinized’ behaviour in dom‐*E* females and the absence of ‘masculinized’ behaviour in co‐*E* females.

Specifically, within our study population, all living as mixed‐sex pairs of conspecifics, dom‐*E* females directed more dominance (i.e., supplants) and aggressive (i.e., cuffs, bites, lunges) behaviour toward their respective male partner than vice versa (dominance: Mann‐Whitney *U* = 13, *n*_1_ = *n*_2_ = 10, *P* = 0.0025 two‐tailed, figure 3A; aggression: Mann‐Whitney *U* = 11, *n*_1_ = *n*_2_ = 10, *P* = 0.0032 two‐tailed, figure 3B). By contrast, compared to their male partner, co‐*E* females showed significantly less dominance behaviour (Mann‐Whitney *U* = 0.5, *n*_1_ = *n*_2_ = 4, *P* = 0.037 two‐tailed; figure 3A) and equal rates of aggression (Mann‐Whitney *U* = 5.5, *n*_1_ = *n*_2_ = 4, *P* = 0.561 two‐tailed; figure 3B). Moreover, within sexes, co‐*E* females expressed significantly reduced rates of dominance behaviour (directed to their partner) than did dom‐*E* females (Mann‐Whitney *U* = 5.5, *n*_1_ = 10, *n*_2_ = 4, *P* = 0.043 two‐tailed; figure 3A).

Supporting the inferences derived from prior chemical data, we also found that dom‐*E* females scent marked just as often as did their male companion (Mann‐Whitney *U* = 48, *n*_1_ = *n*_2_ = 10, *P* = 0.89 two‐tailed, figure 3C), and significantly more so than did co‐*E* females (Mann‐Whitney *U* = 2, *n*_1_ = 10, *n*_2_ = 4, *P* = 0.008 two‐tailed, figure 3C). By contrast, when we examined neutral social behaviour not typically linked to female masculinization, such as allo‐grooming, we found no sex difference in either group (FSD: Mann‐Whitney *U* = 50, *n*_1_ = *n*_2_ = 10, *P* = 0.99; egalitarian: Mann‐Whitney *U* = 7, *n*_1_ = *n*_2_ = 4, *P* = 0.83) and no difference between the females of either group (Mann‐Whitney *U* = 14, *n*_1_ = 10, *n*_2_ = 4, *P* = 0.43). By confirming the lack of intersexual dominance in *E. fulvus* species, as well as describing reduced aggression and scent marking in co‐*E* females, we established two study groups of females from closely related species portraying different degrees of female masculinization, one group with morphological masculinization only, the other group with both morphological and behavioural masculinization.

Next, we compared the hormonal profiles between and, more importantly, within the sexes of our two study groups. We measured serum concentrations of serotonin (5‐HT: 5‐hydroxytryptamine), the androgens A_4_ and T, and the estrogen, estradiol (E_2_: 17β‐estra‐1,3,5(10)‐triene‐3,17‐diol). All four analytes typically reveal measurable sex differences in mammals and have been shown to correlate, particularly in males, either negatively (e.g. 5‐HT) or positively (e.g. androgens and estrogens) with the expression of aggression and dominance^36^.

As anticipated from the adult endocrine patterns observed in *Crocuta*^6^,^30^,^31^,^33^ and *Lemur*^7^,^23^,^29^, both of our *Eulemur* study groups showed the typical mammalian sex difference in T (figure 3D). Relative to co‐*E* females, however, we found a clear, masculine endocrine signature in dom‐*E* females, evident in their significantly greater circulating concentrations of both A_4_ (Mann‐Whitney *U* = 5, *n*_1_ = 10, *n*_2_ = 4, *P* = 0.036 two‐tailed; figure 4A) and T (Mann‐Whitney *U* = 4, *n*_1_ = 10, *n*_2_ = 4, *P* = 0.028 two‐tailed; figure 4B), as well as in their modest trend toward reduced circulating concentrations of 5‐HT (Mann‐Whitney *U* = 9, *n*_1_ = 10, *n*_2_ = 4, *P* = 0.14 two‐tailed, *n.s.*; figure 4C). We also found a trend toward increased circulating concentrations of E_2_ in dom‐*E* females relative to co‐*E* females (Mann‐Whitney *U* = 7, *n*_1_ = 10, *n*_2_ = 4, *P* = 0.076 two‐tailed, *n.s.*; figure 4D). Given that E_2_ derives from the aromatization of androgen precursors, it is likely that the latter trend owes to the raised androgen concentrations of dom‐*E* females. These analyses provide new comparative evidence of ‘masculinized’ androgen profiles in adult females characterized by FSD – a result that would not have been apparent using only between‐sex comparisons.

**Figure 1:**
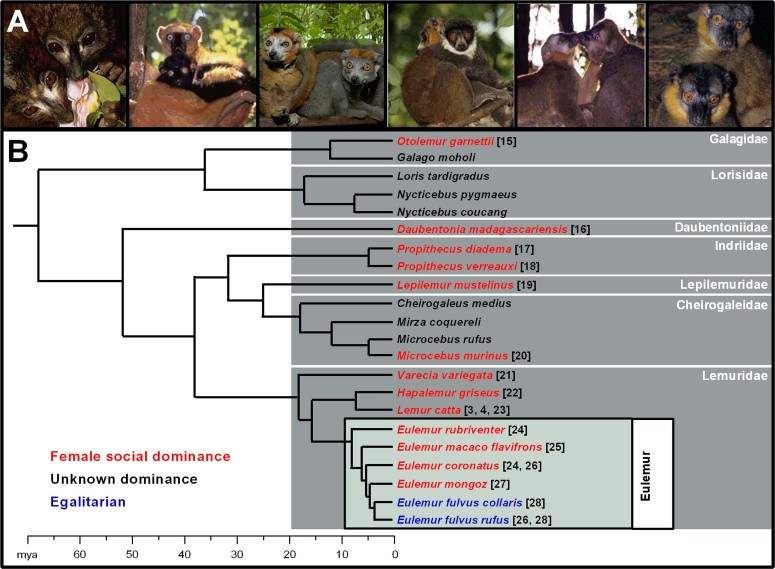
Species within the genus *Eulemur* (A) play a key role in tests of female social dominance or ‘FSD’ (B). In (A), male and female *Eulemur* (shown from left to right as *E. rubriventer*, *E. macaco flavifrons*, *E. coronatus*, *E. mongoz*, *E. fulvus collaris*, and *E. f. rufus*) are sexually dichromatic, but size monomorphic. In (B), FSD (depicted by species shown in red) is widespread across the strepsirrhine lineage (as is female genital masculinization^12^,^13^), and appears to be diminished (depicted by species shown in purple) in only one of the most recently evolved clades of lemur, the *Eulemur* (identified by the box within Lemuridae). In the remaining species (depicted in black), social structure is either unknown or the species tends to be solitary. *Eulemur* photos are reproduced with permission from David Haring, Duke Lemur Center. The phylogeny and estimated divergence times of strepsirrhine primates are adapted from Ref. 14, modified with permission from Cold Spring Harbor Laboratory Press. Refs. 15–28 following species names document FSD.

**Figure 3:**
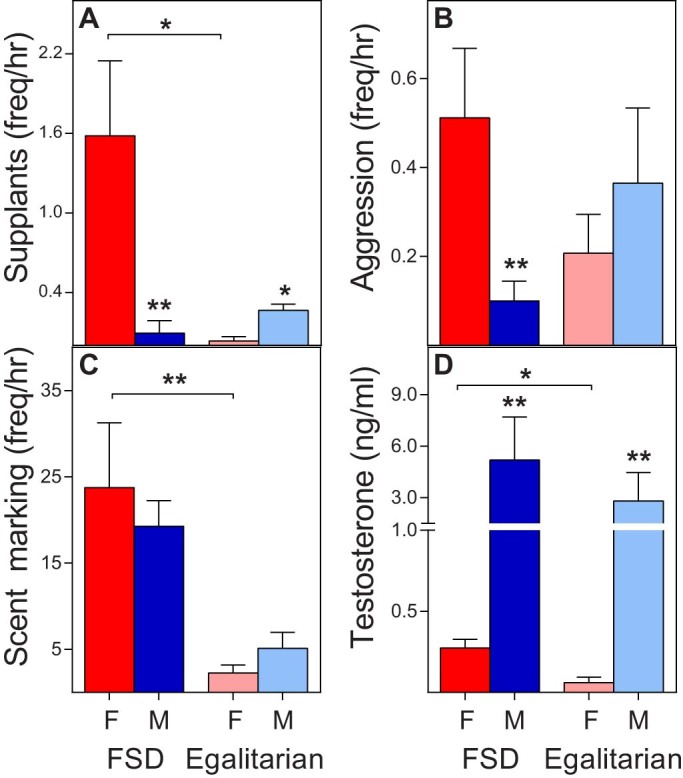
*Eulemur* species expressing female social dominance (‘FSD’) differ markedly in behaviour from closely related species that are egalitarian, but all show the typical mammalian sex difference in testosterone concentrations. Shown are mean + S.E.M. rates of (A) dominance behaviour, (B) aggression, and (C) scent marking, as well as mean + S.E.M. concentrations of (D) serum testosterone (ng/ml) for each member of the mixed‐sex pairs. Female (red/pink); male (blue/light blue). Between sex comparisons (no brackets); between female comparisons (brackets): **P < 0.01 and *P < 0.05.

**Figure 4:**
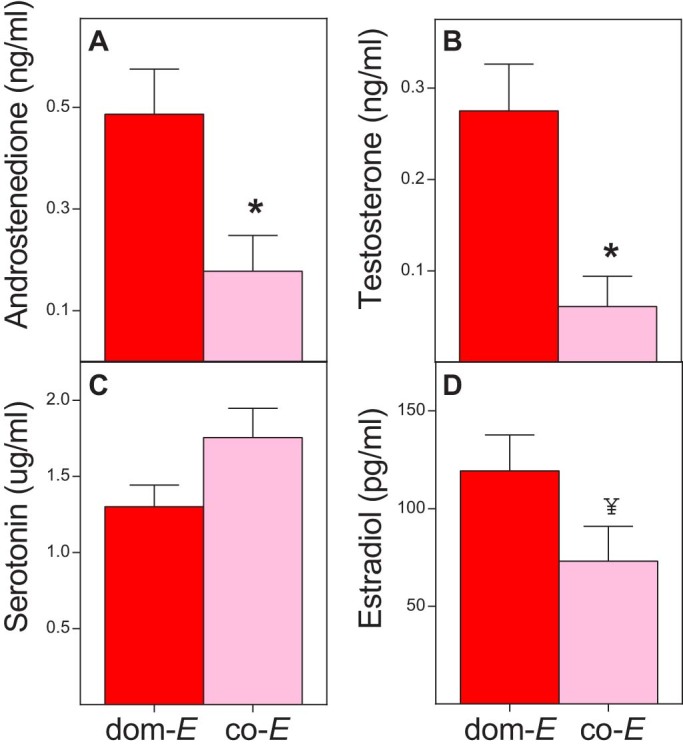
Females of *Eulemur* species characterized by female dominance (dom‐*E*, red) are hormonally ‘masculinized’ relative to females of *Eulemur* species characterized by sexual co‐dominance (co‐*E*, pink). Shown are mean + S.E.M. serum concentrations of (A) androstenedione, (B) testosterone, (C) serotonin, and (D) estradiol. Between female comparisons: *P < 0.05, and ^¥^P < 0.10.

Given the sequential, temporal progression in mammalian sexual differentiation (with hormones affecting first the internal genitalia, then the external genitalia, and finally the brain^8^,^9^,^10^,^11^), masculinization of morphology can be decoupled from masculinization of behaviour via the timing, duration, or dosage of androgen exposure. We propose an evolutionary scenario whereby coupled morphological and behavioural masculinization of females was the ancestral state in strepsirrhines: Given the diversity of species expressing FSD (figure 1) and/or a hypertrophied clitoris^7^,^12^,^13^, female masculinization would have been present minimally 66 MYA, when the lemuriform and lorisiform lineages split^14^. More recently, the behavioural component of masculinization (as well as its purported relation to chemical scent signals^34^) appears to have been diminished. As androgen exposure typically bears a cost^32^,^37^,^38^,^39^, perhaps relaxed intersexual pressures in *E. fulvus* selected for reduced androgen production in adult co‐*E* females and/or in a change in their late‐term hormonal milieu during pregnancy.

As in *Crocuta*^30^,^40^, confirmation of this proposed scenario for the various *Eulemur* species would require either hormonal study of the maternal‐fetal unit or manipulation of the prenatal endocrine milieu, followed by long‐term study of postnatal development. Because this clade includes critically endangered species^5^ whose existence and breeding success in captivity is severely limited, physiological research on pregnant individuals is not presently feasible. Nevertheless, these comparative data provide a rare glimpse into the differing hormone profiles of key species, and strongly support an underlying hormonal mechanism in the evolution of FSD. Further understanding of FSD, in light of the greater androgen concentrations of adult males compared to adult females, may require implicating sex differences in androgen‐receptor distribution or sensitivity, as has been documented recently in other species^41^. Lastly, recognizing both the widespread prevalence of female masculinization across the strepsirrhine lineage and the recent relaxation of FSD in the *E.* *fulvus* clade could transform our understanding of the ancient socioecology and evolution of primate social systems.

## Methods

### Subjects

Our subjects were 28 reproductively intact, adult animals (14 males, 14 females), aged 9–29 (mean + S.E.M.: 20.32 + 0.98) years, representing six species of *Eulemur* (figure 1). The animals were maintained in 14 conspecific, mixed‐sexed pairs at the Duke Lemur Center (DLC) in Durham, NC, USA. All DLC animals carry microchips for identity confirmation and all members of the *Eulemur* colony were individually known and easily recognizable. Moreover, the sexes are sexually dichromatic (figure 1A), further facilitating rapid recognition. The pairs were well established by the time we began our study, as defined by having cohabitated for >1 year. The animals included 10 pairs from four species characterized by FSD and 4 pairs from two species putatively characterized as egalitarian. All of our protocols were performed in accordance with USDA guidelines and were approved by the DLC research committee and the Institutional Animal Care and Use Committee of Duke University (protocols: #MO‐4‐10‐2, A102‐10‐04).

As in *Lemur catta*, the members of the various *Eulemur* species exhibit strictly seasonal estrous cycles, with seasons in the Northern Hemisphere being shifted by six months from those in Madagascar^23^,^29^. Breeding by the *Eulemur* at the DLC generally begins in October, with the peak in births occurring in March and weaning being completed by the end of May. So as to avoid individual variability owing to differences in the timing of female reproductive cycles, we studied the animals across a 3‐month period from June to September 2010, examining behaviour and its endocrine correlates during the nonbreeding season.

### Housing

Our focal pairs of animals were housed in large, indoor/outdoor enclosures (23.2–951.3 m^2^), and were exposed to natural daylight and the local photoperiod. During the warmer months, some of the animals had access to larger, forested enclosures (1.5–27.2 acres), often with several other non‐*Eulemur* species occupying the same habitat. The housing has been described previously^34^. *Eulemur mongoz* are fed folivore chow (Leaf‐Eater Primate Diet, Mazuri, Land O'Lakes Purina Feed, St. Paul, MN, USA), whereas the other *Eulemur* species are fed Old World monkey chow (Monkey Diet, LabDiet, St. Louis, MO, USA). The diets of all the animals are supplemented with a mixture of fruits and vegetables. Those animals that semi‐free‐range can additionally supplement their normal diet with local vegetation and with insects foraged from the forest.

### Behavioural observation

Animals at the DLC are well habituated to the presence of humans, so behavioural observation can occur from short distances (e.g. 1 m), even within the forest. Because each of our focal pairs was housed within a well‐defined area and separately from other *Eulemur*, and because the sexes are readily differentiated even from a distance, we could use continuous focal animal sampling of both dyad members, concurrently. We observed each dyad for 1 hour twice per week in the mornings between 8:30 h and 12:30 h, with the distribution of observation periods being randomized across dyads (for a total of 24 hrs per dyad).

A comprehensive ethogram included dominance behaviour (e.g. supplant), aggression (e.g. lung, bite, chase, cuff), affiliation (e.g. proximity, greet, body contact, allogroom), and scent marking (e.g. deposit, sniff, lick, overmark). We entered the behavioural data, with a time stamp for each event, directly into hand‐held, portable computers (Psion ‘Workabout’, Noldus Information Technology, Inc., Leesburg, VA, USA). We used either an ‘actor‐behaviour’ format (for scent marking) or an ‘actor‐behaviour‐recipient’ format (for dominance, aggression, and affiliation), which allowed us to record the frequency, directionality, and duration of all interactions between the male and female pair members. All four observers were trained prior to data collection and were tested routinely for inter‐observer reliability (mean ± S.D. = 88.7 ± 0.1%). To further minimize the effect of variation in the data potentially owing to multiple observers, each dyad was observed an equal number of times by each observer over the course of the study.

### Sampling procedures

With the assistance of DLC veterinary personnel, we obtained from each subject one blood sample per month, for a total of three samples per subject in the nonbreeding season (total *n* = 42 male and *n* = 42 female samples). On blood‐draw days, the animals that previously had been corralled into their indoor enclosures were netted, transported to a veterinary procedure room located in the same building, and processed individually, to minimize the time delay between capture and blood draw (mean ± S.E.M. = 5.00 ± 0.68 min). Handling occurred primarily in the morning (between 9:00 and 12:30 h, mean ± S.E.M. = 10:12 ± 0:07 h). Using a 23‐gauge needle and syringe, we drew blood samples (3 cc) from the femoral vessels of awake, manually restrained animals, all of which were habituated to these procedures. We immediately transferred the blood samples to serum separator tubes (Vacutainer®, Becton Dickinson, Franklin Lakes, NJ, USA), allowed them to clot at room temperature, centrifuged them at 1500 × g for 20 min, and stored the decanted serum at −80°C.

### Hormone assays

We determined serum concentrations of 5‐HT, A_4_ and T, and E_2_ using commercial, competitive enzyme immunoassay (EIA) kits (ALPCO diagnostics, Salem, NH, USA). We validated the EIA assays for analyte recovery by spiking a known amount of analyte into a pooled serum sample and comparing the observed and expected results. We validated the EIA assays for linearity by running a serial dilution of the pooled serum and comparing the slopes against the standard curves. For all assays, recovery ranged from 85% to 105% and each dilution curve was parallel to the appropriate assay standard curve. The 5‐HT assay has a sensitivity of 5 ng/ml using a 25‐μl dose, with an intra‐ and inter‐assay coefficient of variation (CV) of 5.4% and 6%, respectively. The A_4_ assay has a sensitivity of 0.04 ng/ml using a 25‐μl dose, with an intra‐ and inter‐assay CV of 5.23% and 8.7%, respectively. The T assay has a sensitivity of 0.02 ng/ml using a 50‐μl dose, with an intra‐ and inter‐assay CV of 7.9% and 7.3%, respectively. The E_2_ assay has a sensitivity of 10 pg/ml using a 50‐μl dose, with an intra‐ and inter‐assay CV of 7.7% and 8.7%, respectively. We performed all assays in duplicate. If any individual sample's duplicate CV exceeded the intra‐assay CV it was re‐run in a subsequent assay.

### Statistical analyses

We averaged each individual's data points across the study. In the event that an individual's assay result was below the level of detectability, we used the minimum sensitivity value for that assay in our calculations. We tested for sex effects within each study group or type of dominance structure (e.g. female FSD vs. male FSD) and for effects of dominance structure within the female sex (i.e., dom‐*E* vs. co‐*E*). We conducted these analyses separately for both the behavioural and endocrine data. To account for small sample sizes and lack of normality in the data, we used the two‐tailed Mann‐Whitney U test for all comparisons.

## Author Contributions

C.M.D. conceived of and conducted the comparative strepsirrhine work on female dominance, genital masculinization, and olfactory communication. J.M.A.P. designed and conducted the comparative *Eulemur* research under the supervision and guidance of C.M.D. J.M.A.P. analyzed the data and prepared the figures. C.M.D. and J.M.A.P. wrote the manuscript.

## Figures and Tables

**Figure 2:**
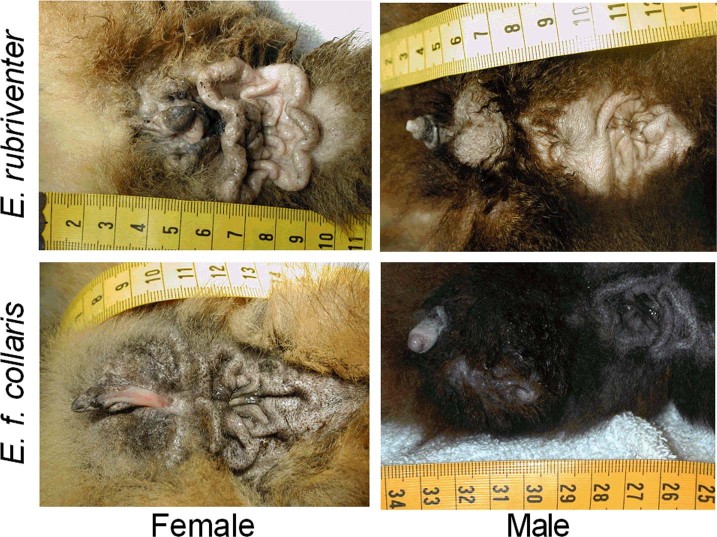
Across *Eulemur* species, females appear to be equally ‘masculinized’ in their morphological features, in that all females have an elongated, pendulous clitoris that is partially traversed by the urethra, and their peri‐anal glands are more elaborate than are those of conspecific males. Pictured are the anogenital regions (in cephalocaudal orientation from left to right) of representative, adult female (left column) and male (right column) members of *E. rubriventer* (top row), a species characterized by female social dominance, and *E. f. collaris* (bottom row), a species characterized by egalitarianism.
